# Evaluation of the Function of the ASFV *KP177R* Gene, Encoding for Structural Protein p22, in the Process of Virus Replication and in Swine Virulence

**DOI:** 10.3390/v13060986

**Published:** 2021-05-26

**Authors:** Elizabeth A. Vuono, Elizabeth Ramirez-Medina, Sarah Pruitt, Ayushi Rai, Nallely Espinoza, Lauro Velazquez-Salinas, Douglas P. Gladue, Manuel V. Borca

**Affiliations:** 1 Plum Island Animal Disease Center, Agricultural Research Service, United States Department of Agriculture Greenport, Greenport, NY 11944, USA; Elizabeth.Vuono@usda.gov (E.A.V.); elizabeth.ramirez@usda.gov (E.R.-M.); Sarah.Pruitt@usda.gov (S.P.); ayushi.rai@usda.gov (A.R.); Nallely.Espinoza@usda.gov (N.E.); Lauro.Velazquez@usda.gov (L.V.-S.); 2 Department of Pathobiology and Population Medicine, Mississippi State University, Starkville, MS 39762, USA; 3 Oak Ridge Institute for Science and Education (ORISE), Oak Ridge, TN 37830, USA; 4 Department of Anatomy and Physiology, Kansas State University, Manhattan, KS 66506, USA

**Keywords:** ASFV, ASF, African swine fever virus, KP177R, p22

## Abstract

African swine fever virus (ASFV) causes a devastating disease of swine that has caused outbreaks in Central Europe since 2007, spreading into Asia in 2018. ASFV is a large, structurally complex virus with a large dsDNA genome encoding for more than 160 genes, most of them still uncharacterized. p22, encoded by the ASFV gene KP177R, is an early transcribed, structural virus protein located in the ASFV particle. Although its exact function is unknown, p22 has recently been identified as an interacting partner of several host proteins. Here, we describe the development of a recombinant ASFV (ASFV-G-∆KP177R) lacking the KP177R gene as a tool to evaluate the role of p22 in virus replication and virulence in swine. The recombinant ASFV-G-∆KP177R demonstrated that the KP177R gene is non-essential for ASFV replication in primary swine macrophages, with virus yields similar to those of the parental, highly virulent field isolate Georgia2010 (ASFV-G). In addition, experimental infection of domestic pigs with ASFV-G-∆KP177R produced a clinical disease similar to that caused by the parental ASFV-G. Therefore, and surprisingly, p22 does not seem to be involved in virus replication or virulence in swine.

## 1. Introduction

African swine fever virus (ASFV) is causing a pandemic affecting a contiguous geographical region extending from central Europe to China and into Southeast Asia, causing a potential worldwide shortage of protein availability and economic losses to local and national swine industries [1]. 

ASFV is a structurally complex enveloped virus harboring a large (180–190 kilobase pairs), double-stranded DNA genome encoding for over 150 different genes [2]. No commercial vaccines are available to prevent African swine fever (ASF); therefore, the control of disease outbreaks involves culling susceptible animals at infected farms and implementing strict biosecurity measures to prevent disease spread to uninfected farms. 

ASFV experimental vaccines developed through the deletion of specific genes from the virus genome have been shown to be effective in protecting against the current circulating strain in Europe and Asia [3,4,5,6,7,8]. The development of those vaccines was possible by identifying and characterizing virus genes involved in the process of virus virulence, highlighting the importance of understanding the role of individual genes and how their manipulation could be used to develop experimental vaccines. 

ASFV encodes for more than 150 genes, of which few are experimentally characterized [1], with the role of most ASFV genes remaining largely unknown [2]. Understanding the role of viral proteins in the process of virus replication and/or virus virulence is critical to developing novel countermeasures for disease control. The discovery of ASFV gene function using genetic manipulation techniques has resulted in several experimental ASFV live-attenuated vaccines [3,4,5,6,7,8]. Only a small number of virus genes have been successfully deleted from the ASFV genome, producing novel deletion mutants of the virus (e.g., TK, NL, CD2, MGF360-16R and 1L, MGF110-1L, L83L, C962R, X69R, and I8L) [9,10,11,12,13,14,15,16,17,18], and another small number of genes determined to be essential for virus replication (e.g., EP152R, p30, p54, and p72) [19,20,21,22]. Deleting specific genes by genetic manipulation of the virus genome is an extraordinarily powerful approach to study the function of a particular gene during virus–cell interactions. 

The KP177R gene encodes for the virus protein p22. p22 was originally described as an early transcribed, viral structural transmembrane protein [23]. Further studies locate p22 to the inner membrane of the virus particle and at the surface of infected cells [24]. Recently, it has been shown that ASFV p22 interacts with several host partners involved in different cell pathways contributing to endocytosis, the cyclic GMP-dependent protein kinase (cGMP-PKG) signaling pathway, the cAMP signaling pathway, and the AMP-activated protein kinase (AMPK) signaling pathway [25]. These interactions suggest p22 could be involved in several critical functions during ASFV replication in vitro and in vivo. The main aim of this report was to understand the importance of p22 during ASFV replication in swine macrophage cultures and during experimental infection in domestic pigs. 

## 2. Materials and Methods 

### 2.1. Viruses and Cells 

Primary swine macrophage cell cultures were prepared from swine blood as previously described in detail [26]. Peripheral blood mononuclear cells were purified by a Ficoll-Paque (Pharmacia, Piscataway, NJ, USA) density gradient and cultured for 24 h at 37 °C under 5% CO_2_. Adherent cells were detached from the Primaria flasks and reseeded into Primaria T25, 6- or 96-well dishes at a density of 5 × 10^6^ cells per ml for use in assays 24 h later. ASFV Georgia (ASFV-G) was a field isolate kindly provided by Nino Vepkhvadze from the Laboratory of the Ministry of Agriculture (LMA) in Tbilisi, Republic of Georgia [10]. 

Comparative growth curves between ASFV-G-∆KP177R and parental ASFV-G were performed in primary swine macrophage cell cultures in 24-well plates and were infected at an MOI of 0.01 (based on HAD_50_ (50% hemadsorption dose), previously determined in primary swine macrophage cell cultures). The initial inoculum was removed after adsorption for 1 h at 37 °C under 5% CO_2_. Cells were then rinsed with PBS twice and incubated with macrophage media for 2, 24, 48, 72, and 96 h at 37 °C under 5% CO_2_. At these times post-infection, the cells were frozen at ≤−70 °C, and the thawed lysates were used to determine titers by HAD_50_/mL in primary swine macrophage cell cultures in 96-well plates. All samples were run simultaneously to avoid inter-assay variability. The presence of the virus was assessed by hemadsorption (HA), and virus titers were calculated as previously described [27].

### 2.2. Construction of the KP177R Deletion Mutant ASFV 

ASFV lacking the KP177R gene (ASFV-G-∆KP177R) was generated by homologous recombination between the parental ASFV genome and a recombinant transfer vector following previously described procedures [3]. The recombinant transfer vector (p72-mCherryΔKP177R) contained flanking genomic regions of the KP177R gene: the left arm is located between the genomic positions 3157–4157, and the right arm is located between the genomic positions 4742–5742 and harbors a reporter gene cassette containing the fluorescent protein gene (mCherry) under the control of the ASFV p72 late gene promoter [28]. The recombinant transfer vector was obtained by DNA synthesis (Epoch Life Sciences, Sugar Land, TX, USA). As designed, this construction created a 571-nucleotide deletion between nucleotide positions 4171–4741, completely deleting the KP177R ORF sequence. Recombinant mutant ASFV-G-∆KP177R was purified to homogeneity by successive rounds of limiting dilution purification, using the highest dilution with detectable amounts of mCherry. The full length of the ASFV DNA, extracted from infected cells, was sequenced using next-generation sequencing (NGS) as previously described [28] with an Illumina NextSeq500 sequencer. Sequence analysis was performed using CLC Genomics Workbench software version 20 (QIAGEN, Hilden, Germany). .

### 2.3. Animal Experiments

Virulence of ASFV-G-∆KP177R was evaluated using 35–40 kg of commercial breed swine. Five pigs were inoculated intramuscularly (IM) with 10^2^ HAD_50_ of ASFV-G-∆KP177R and compared with a group of pigs (n = 5) inoculated with 10^2^ HAD_50_ of ASFV-G. Clinical signs (anorexia, depression, fever, purple skin discoloration, staggering gait, diarrhea, and cough) and changes in body temperature were recorded daily throughout the experiment. Blood samples were obtained at 0, 4, and 7 days post-inoculation (pi). Animal experiments were performed under biosafety level 3 conditions in the animal facilities at Plum Island Animal Disease Center, following a strict protocol approved by the Institutional Animal Care and Use Committee (225.01-16-R approved on 09-07-16).

## 3. Results and Discussion

### 3.1. KP177R Gene Is Conserved Across Different ASFV Isolates

The KP177R gene encodes for virus protein p22, originally described as an early transcribed transmembrane protein that is associated with the outer layer of the virus particle, as well as being transiently expressed on the surface of infected cells [23]. Recent studies have located p22 to the inner membrane of the virus particle [24].

**Figure 1:**
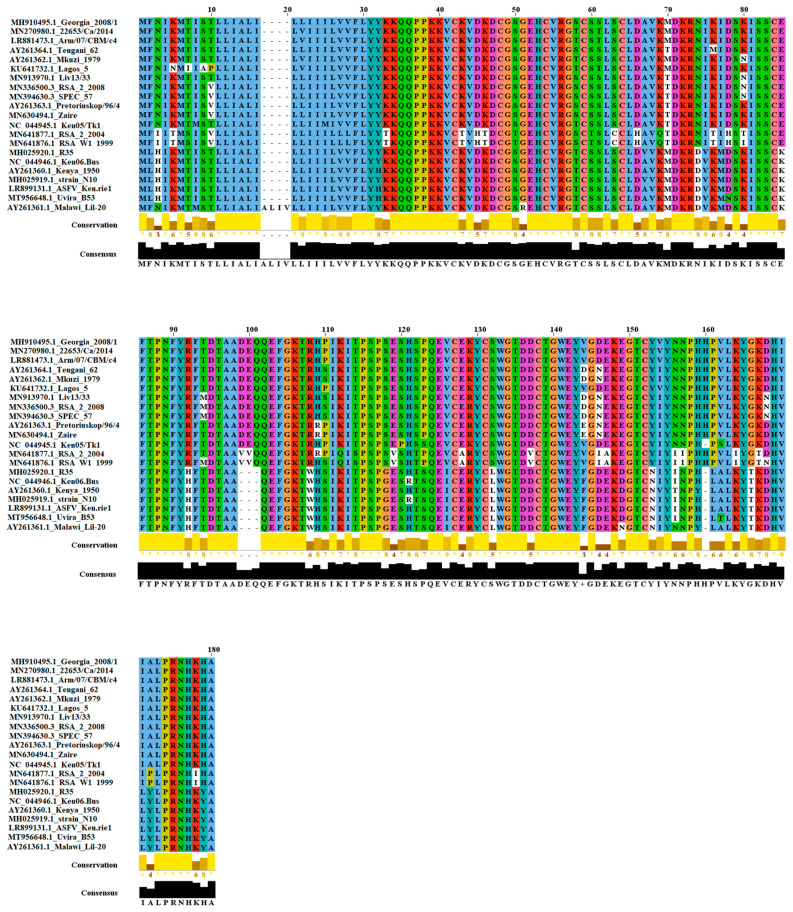
Amino acid diversity of protein p22. Twenty-one protein sequences representing the amino acid diversity of protein p22 (KP-177-R gene) of ASFV within the GenBank database were used to conduct this alignment. To assess the nature of the replacements at multiple residues, conservation scores based on the biological properties of each amino acid were included, the lower scores being associated with more divergent replacements. Symbols (*) indicate residue conservation or (+) replacement for an amino acid with similar properties. Analysis was conducted on Jalview software version 2.11.1.3, using the ClustalW algorithm sequence alignment of the indicated ASFV isolates of viral protein PK177R. Matching residues are represented as dots. The degree of conservation is below the alignment.

To evaluate the nucleotide and amino acid conservation across different isolates of ASFV representing the genetic diversity of gene KP177R, we developed alignments using ClustalW. Nucleotide homology varied between 84.81% and 99.81%, and amino acid homology varied between 66.86% and 99.41%, with an average nucleotide homology of 92.86% and an average amino acid homology of 86.94% (Figure 1).

To further investigate the disparate range of homology of p22, we performed specific pairwise calculations between isolates. In general, we observed that the disparate range of conservation at the amino acid level was found in the comparison of isolates RSAW1/1999 and RSA2/2004, a group of viruses isolated from South Africa classified as genotypes IV and XX, respectively. In this context, when these isolates were compared with Malawi Lil-20/1, the amino acid conservation was determined to be as low as 64.44%. Conversely, a high level of amino acid conservation (96.02%) was observed when pairwise comparisons were performed between Georgia 2008/1 and Mazuki 1979 isolates.

Interestingly, no differences at nucleotide and amino acid levels were found within all the contemporary Eurasian isolates considered in the analysis, confirming a high degree of conservation within the p22 protein in this lineage, and suggesting a lack of selective pressure on the KP177R gene during the evolution of this lineage, despite more than 10 years of circulation.

### 3.2. Development of the ASFV-G-ΔKP177R Deletion Mutant

The relatively high level of conservation of KP177R among ASFV isolates, due to it being a structural protein [23,24], and recent results showing the interaction of ASFV p22 with host partners involved in several cellular pathways [25], suggests that p22 may be involved in critical virus replication functions.

**Figure 2:**
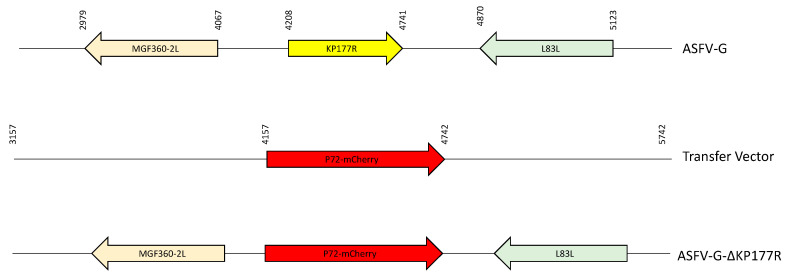
Schematic for the development of ASFV-G-∆KP177R. The transfer vector contains the p72 promoter and an mCherry cassette; the flanking left and right arms are indicated and were designed to have flanking ends to both sides of the deletion/insertion cassette. The nucleotide positions of the ASFV-G genome are indicated. The resulting ASFV-G-∆KP177R virus with the cassette inserted is shown at the bottom.

To study the function of the KP177R gene during ASFV replication in cell cultures and the process of virulence in swine, a recombinant deletion mutant of the highly virulent ASFV Georgia 2007 isolate (ASFV-G) lacking the KP177R gene was produced (ASFV-G-∆KP177R). Deletion of the KP177R gene was achieved by substituting the complete KP177R ORF of 177 amino acid residues with a p72-mCherry cassette by homologous recombination [28]. A region spanning 571 bp (between nucleotide positions 4171 and 4741) was deleted from the ASFV-G genome in order to delete the entire Kp177R gene, including the potential start site described in the original Georgia 2007/1 annotation [29], which was later deemed to be out of frame with the KP177R gene when Georgia 2007/1 annotation was reviewed [30] and substituted with a 1226 bp cassette containing the p72-mCherry construct (see Material and Methods) (Figure 2). ASFV-G-∆KP177R stock was purified after successive limiting dilution steps using primary swine macrophage cell cultures. The stock virus was produced by amplifying virus obtained from the last purification round in primary swine macrophage cell cultures.

To evaluate the accuracy of genetic modifications of ASFV-G-∆KP177R and the integrity of the remaining virus genome, the full genome sequence was obtained by NGS using an Illumina NextSeq^®^ 500. The comparative analysis of the genomes of ASFV-G-∆KP177R and ASFV-G verified a deletion of 571 nucleotides, which is consistent with the designed genomic modifications. In addition, the genome of ASFV-G-∆KP177R harbors an insertion of 1226 nucleotides consistent with the insertion of the p72-mCherry cassette sequence. No other genomic differences were detected between ASFV-G-∆KP177R and ASFV-G, confirming that no other changes developed during the process of creation and purification of ASFV-G-∆KP177R. In addition, NGS also demonstrated the absence of the residual parental ASFV-G genome as a potential contaminant in the stock of ASFV-G-∆KP177R.

### 3.3. Replication of ASFV-G-ΔKP177R in Primary Swine Macrophages

**Figure 3:**
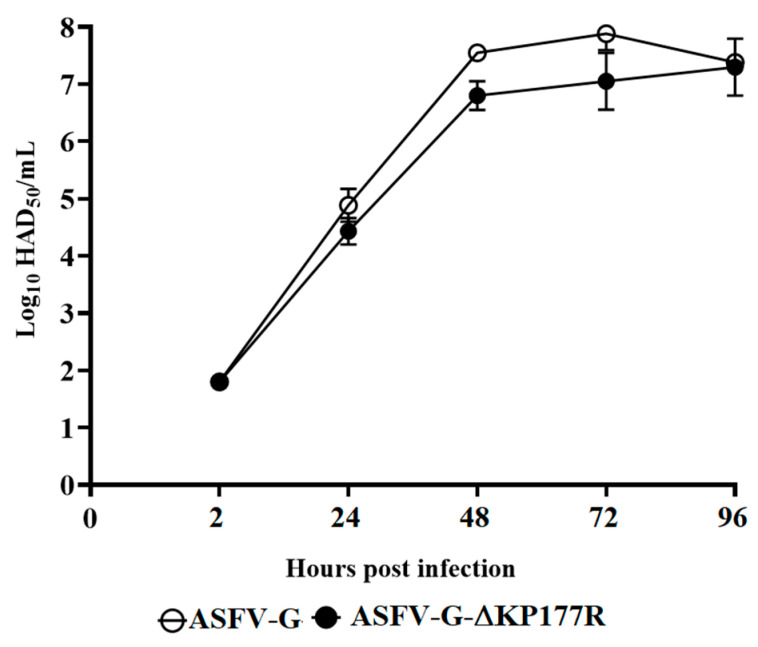
In vitro growth kinetics in primary swine macrophage cell cultures for ASFV-G-∆KP177R and parental ASFV-G (MOI = 0.01). Samples were taken from three independent experiments at the indicated time points and titrated. Data represent means and standard deviations. Sensitivity using this methodology for detecting the virus is >log_10_ 1.8 HAD_50_/mL. No significant differences in viral yields between viruses were observed at any time point tested as determined using the Holm–Sidak method (α = 0.05), without assuming a consistent standard deviation. All calculations were conducted using Graphpad Prism software version 8.

To investigate the potential role of KP177R during virus replication, the in vitro growth kinetics of ASFV-G-∆KP177R were assessed in comparison to that of the parental ASFV-G in a multi-step growth curve using swine macrophage cultures as a substrate. Macrophage cultures were infected at an MOI of 0.01 with either ASFV-G-∆KP177R or ASFV-G, and samples needed to evaluate virus yield were collected at 2, 24, 48, 72, and 96 h post-infection (pi). The results demonstrated that ASFV-G-∆KP177R displayed a very similar growth kinetic to that of the parental ASFV-G without significant differences in virus yields at any of the evaluated times post-infection (Figure 3). Therefore, deletion of the KP177R gene from the genome of ASFV-G appears to not significantly affect the ability of the virus to replicate in swine macrophages. This is a surprising result, considering the fact that the gene is somewhat conserved across all known ASFV genomes and that the encoded protein, p22, has been detected as part of the virus particle [23,24] and is described to interact with several host cell ligands [25].

### 3.4. Assessment of ASFV KP177R Virulence in Swine

**Figure 4:**
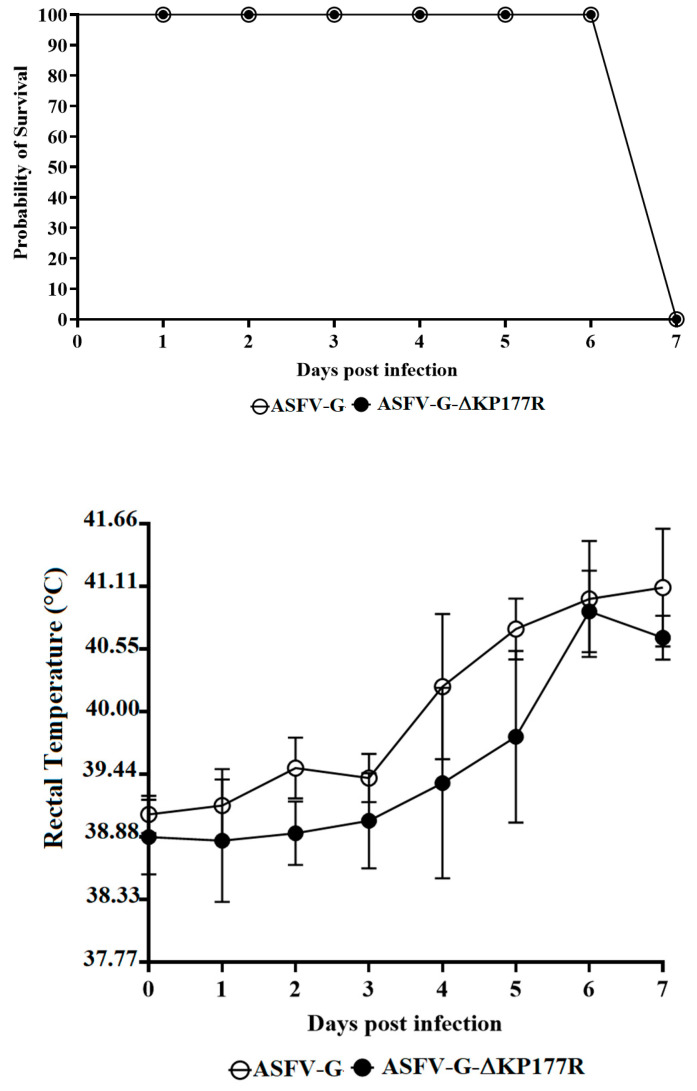
Evolution of mortality (**top panel**) and body temperature (**bottom panel**) in animals (5 animals/group) IM infected with 10^2^ HAD_50_ of either ASFV-G-∆KP177R (filled symbols) or parental ASFV-G (open symbols). Significant differences (*p* value = 0.0201) in the survival course between groups of pigs were found using the log-rank test (Mantel–Cox test). No statistical differences were found in body temperatures between pigs in both groups when evaluated by the Holm–Sidak method (α = 0.05). All calculations were conducted using GraphPad Prism software version 8.

To evaluate the effect of the KP177R gene deletion on the virulence of ASFV-G, a group of domestic pigs were IM inoculated with 10^2^ HAD_50_ per animal. An additional control group was also IM inoculated but with 10^2^ HAD_50_ of the parental ASFV-G. All animals inoculated with virulent ASFV-G, as expected, had an initial increase in body temperature (>104 °F) by days 4–5 pi, followed by the rapid development of clinical signs associated with the disease (depression, anorexia, staggering gait, diarrhea, and purple skin discoloration) (Table 1 and Figure 4). The clinical disease quickly aggravated; therefore, all animals needed to be euthanized in extremis by day 7 pi.

Interestingly, animals inoculated with ASFV-G-∆KP177R developed a clinical disease similar to that present in animals inoculated with parental ASFV-G. Kinetics of presentation of clinical signs as well as their severity resembled those present in animals inoculated with ASFV-G. The presentation of these indistinguishable clinical signs between the two groups of animals suggests that deletion of the KP177L gene from the genome of the highly virulent isolate ASFV-G does not affect virus virulence in domestic swine.

**Figure 5:**
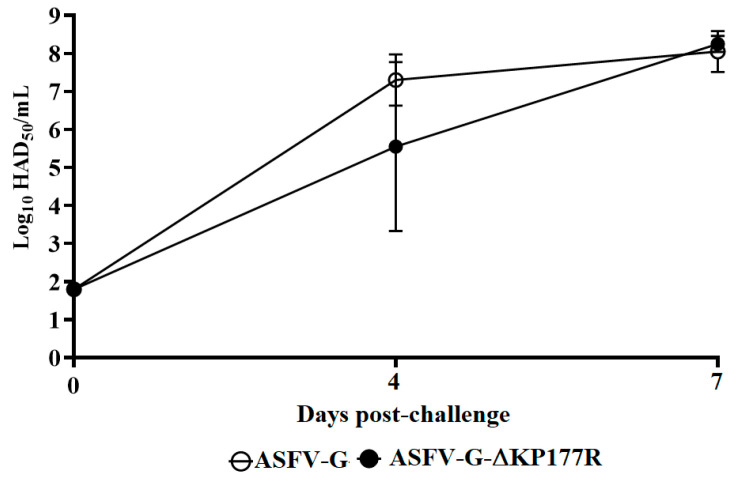
Viremia titers detected in pigs IM inoculated with 10^2^ HAD_50_ of either ASFV-G-∆KP177R (filled symbols) or ASFV-G (empty symbols). Each symbol represents the average of animal titers in each of the groups. Sensitivity of virus detection: >log_10_ 1.8 TCID_50_/ml. Significant differences in viremia values between both groups of pigs were found at day four post-infection using the Holm-Sidak method (α = 0.05) without assuming a consistent standard deviation. All calculations were conducted on the software GraphPad Prism version 8.

Systemic virus replication in animals was assessed by determining viremia titers throughout the experimental period. Viremias in animals IM infected with parental ASFV-G had expected high titers (10^6.5^–10^7.5^ HAD_50_/mL) on day 4 pi, remaining high until day 7 pi, when all animals were euthanized. All animals inoculated with ASFV-G-∆KP177R had viremia values ranging from 10^3^–10^6^ HAD_50_/ml by day 4 pi, reaching maximum titers by day 7 pi, when all animals were euthanized (Figure 5). Therefore, only statistical differences were transiently found in the average of viremia titers at 4 dpi, while at 7 dpi, viremia titers were indistinguishable between animals inoculated with either virus.

These results would imply that deletion of KP177R from the genome of ASFV-G does not significantly affect the process of virus replication or virulence in domestic swine. To further confirm that ASFV-G-∆KP177R was responsible for the virulent phenotype and viremia levels observed, the virus was isolated from the blood of ASFV-G-∆KP177R-infected animals and analyzed by NGS. Results obtained by sequencing samples from three animals demonstrated the absence of any significant differences with the full-length genomic nucleotide sequence of the ASFV-G-∆KP177R stock.

In summary, we determined that KP177R is a non-essential gene since its deletion from the ASFV-G genome does not significantly alter virus replication in vitro, in swine macrophage cultures, or during infection in vivo and, importantly, is not critical for ASFV virulence in swine. It was unexpected that structural protein p22 is apparently not involved in basic critical virus functions, at least in those that were tested here. An explanation would be the potential replacement of the KP177R gene function by one of the L101L genes located in the right end of the virus genome. The L101L and KP177R genes showed a medium-to-high level of amino acid identity among different ASFV isolates [29]; therefore, it was hypothesized that there is a potential overlapping in the function of these two genes. Some of the ASFV structural proteins, such as p72 or p54, are essential for virus viability [22,23] and cannot be removed from the virus genome. Conversely, deletion of the EP402R gene, encoding for the ASFV CD2-like gene, another ASFV structural protein, does not affect virus replication in cell cultures or in vivo, nor does it alter virus virulence [6,11].

## Figures and Tables

**Table 1:** Swine survival and fever response following infection with ASFV-G-∆KP177R and parental ASFV-G.

			Fever		
Virus (10^2^ HAD_50_)	No. of Survivors/Total	Mean Time to Death (±SD)	No. of Days to Onset (±SD)	Duration No. of Days (±SD)	Maximum Daily Temp, °C (±SD)
ASFV-G-ΔKP177R	0/5	7 (0)	5 (1)	2 (1)	40.89 (0.24)
ASFV-G	0/5	7 (0)	4.2 (0.45)	2.8 (0.45)	41.06 (0.95)

## Data Availability

Data is contained within the article.
